# Automatic number priming effects in adults with and without mathematical learning disabilities

**DOI:** 10.3389/fpsyg.2014.00004

**Published:** 2014-02-05

**Authors:** Emmy Defever, Silke M. Göbel, Pol Ghesquière, Bert Reynvoet

**Affiliations:** ^1^Experimental Psychology, KU LeuvenLeuven, Belgium; ^2^Faculty of Psychology and Educational Sciences, KU Leuven KulakKortrijk, Belgium; ^3^Department of Psychology, University of YorkYork, UK; ^4^Parenting and Special Education Research Unit, Faculty of Psychology and Educational Sciences, KU LeuvenLeuven, Belgium

**Keywords:** mathematical learning disabilities, magnitude representation, priming task, access deficit, representation deficit

## Abstract

This study examined automatic number processing in adults with mathematical learning disabilities (MLDs). The performance of adults with MLD during an automatic symbolic and non-symbolic priming task was compared to gender-, age-, and IQ-matched controls. No difference in the priming distance effect was found between the adults with and without MLD, suggesting that adults with MLD have an intact magnitude representation. Moreover, the adults with MLD did not have problems in processing the numerical symbols 1–9, suggesting that this basic deficit which is experienced by children with MLD is resolved by adulthood.

## INTRODUCTION

Sufficient basic mathematical competence has shown to be fundamental for success in daily life (Duncan et al., 2007; Reyna et al., 2009). There are, however, many individuals struggling with mathematics. Therefore, a thorough understanding of the nature of mathematical learning disabilities (MLDs) is crucial in order to develop appropriate intervention strategies. MLD is generally defined as a specific learning disorder in the domain of mathematics, which must not be caused by sensory difficulties, general intellectual impairment or lack of educational opportunity (American Psychiatric Association, 2000; see Von Aster and Shalev, 2007 for a review). Studies have demonstrated that various cognitive deficits might underpin MLD (Wilson and Dehaene, 2007; Ansari, 2008; Rubinsten and Henik, 2009). On the one hand, domain-general deficits are proposed, such as an impairment of working memory (McLean and Hitch, 1999; Passolunghi and Siegel, 2001; Geary, 2004, 2005), long-term memory (Geary et al., 2000), visuospatial abilities (Rourke and Conway, 1997), executive functioning (Bull and Scerif, 2001) and attention (Ashkenazi et al., 2009). On the other hand, a domain-specific deficit in how numerical magnitudes are represented and processed has been suggested to cause MLD (Butterworth, 2005; Rousselle and Noël, 2007; Wilson and Dehaene, 2007). Concerning this latter account, two main hypotheses have been put forward. Firstly, ***the representation deficit hypothesis*** suggests that MLD is caused by a deficit in the innate ability to mentally represent and process numerical magnitudes, resulting in difficulties in learning about numbers and arithmetic (Dehaene, 1997; Butterworth, 2005). Evidence for this representation deficit hypothesis has mainly been accumulated in children using magnitude comparison tasks (e.g., Landerl et al., 2009; Mussolin et al., 2010b; see De Smedt et al., 2013 for a review). For instance, Mussolin et al. (2010b) found that 10- to 11-year-old children with mathematical difficulties showed a larger distance effect (i.e., longer response times and higher error rates when comparing magnitudes that are numerically close to each other) than controls in both symbolic and non-symbolic magnitude comparisons. This distance effect is usually explained by overlapping representations of nearby magnitudes. A particular magnitude does not only activate its corresponding representation, but also to a lesser extent the representations of numerically close magnitudes, according to a Gaussian distribution (Moyer and Landauer, 1967; Restle, 1970). Accordingly, it is more difficult to discriminate between magnitudes that are numerically close as there is more representational overlap between them. The larger numerical distance effect found in children with mathematical difficulties might suggest that they have a more imprecise representation of number magnitude (Mussolin et al., 2010b). To the best of our knowledge, only few studies addressed this hypothesis in adults (Furman and Rubinsten, 2012; Mejias et al., 2012). Mejias et al. (2012) showed that adults with MLD produced less accurate and more variable estimates than control participants during both symbolic and non-symbolic numerical estimation tasks, suggesting they have a less precise magnitude representation. Additional support for the representation deficit hypothesis comes from cognitive neuroscience. Several neuroimaging studies have demonstrated the pivotal role of the intraparietal sulcus (IPS) in magnitude processing of symbolic and non-symbolic stimuli in children and adults (e.g., Piazza et al., 2007; Notebaert et al., 2011). Atypical activation of the IPS has been found during the comparison of non-symbolic (Price et al., 2007) and symbolic magnitudes (Mussolin et al., 2010a) in children with MLD (but see Kucian et al., 2006; Davis et al., 2009; Kovas et al., 2009). Also structural abnormalities (i.e., reduced gray matter) have been found in this brain area for children with MLD (Rotzer et al., 2008).

Secondly, ***the access deficit hypothesis*** states that MLD does not result from an inability to correctly represent magnitudes, but rather from a deficit in linking a symbol (e.g., Arabic digits) with its intact magnitude representation (e.g., Rousselle and Noël, 2007; Landerl and Kölle, 2009; De Smedt and Gilmore, 2011). For instance, it has been found that children with MLD perform worse compared to controls in symbolic comparison tasks, but not when they have to compare non-symbolic magnitudes (Rousselle and Noël, 2007; De Smedt and Gilmore, 2011). It is not yet clear which brain regions support the linkage between symbols and their number meaning. Recent studies within the field of numerical cognition suggest that intact functioning of the left angular gyrus subserves the connection between symbols and their number meaning (Grabner et al., 2007; Holloway et al., 2010; Price and Ansari, 2011). Up till now, no studies have investigated directly the access deficit hypothesis at the neuronal level in individuals with MLD.

To date, especially comparison and estimation tasks have been used to investigate these hypotheses. Moreover, few studies have tested the hypotheses in adults with MLD (Furman and Rubinsten, 2012; Mejias et al., 2012). In this study, we used an automatic priming paradigm to contrast both hypotheses with the same paradigm. To the best of our knowledge, this paradigm has not yet been previously used to study magnitude processing in participants with MLD. In the priming paradigm, two magnitudes are presented consecutively, the prime and the target. Responses are characterized by the priming distance effect (PDE), indicating faster reaction times (RTs) on trials with numerically close prime-target pairs (e.g., “1” preceded by “2”) than on trials with numerically more distant prime-target pairs (e.g., “1” preceded by “4”). Similar to the comparison distance effect, this PDE is explained by overlapping representations of numerical magnitude. The prime not only activates its own representation but also the representation of magnitudes nearby. Therefore, in close prime-target pairs, the prime already partially activated the representation of the target, which makes it easier to respond to the target than when the distance between prime and target is larger. Hence, the PDE is considered as a useful measure to examine the characteristics of the magnitude representation (Reynvoet and Brysbaert, 1999; Van Opstal et al., 2008; Notebaert et al., 2010). In the present study we conducted a symbolic and non-symbolic priming task. We used a short interval between the onset of the prime and that of the target (i.e., stimulus onset asynchrony; SOA) because it has been suggested that when a short SOA is used, the effects of priming on participant’s responses reflect more automatic processes compared to larger SOAs: strategic expectancy effects (i.e., participants generate expectations about the target based on the prime) require SOAs of more than 250 ms to influence the results (Neely, 1991; Stolz et al., 2005). Our hypotheses were clear-cut. If MLD is related to an inability to represent and process magnitudes (i.e., representation deficit hypothesis), we would expect to observe a smaller PDE in participants with MLD compared to controls in both symbolic and non-symbolic conditions. For instance, less precise magnitude representations lead to a smaller PDE because the representational overlap that is responsible for the PDE will not differ a lot between the different prime-target distances, leading to less increase in RTs with increasing numerical distance. In contrast, if MLD results from a deficit in accessing magnitude information from symbols (i.e., access deficit hypothesis), we would expect that participants with MLD are slower and/or make more errors than controls in the symbolic task only. Because this hypothesis does not make explicit assumptions regarding a deficient representation, no differences in the PDE are expected.

## MATERIALS AND METHOD

### PARTICIPANTS

Sixteen university students with MLD and sixteen gender, age and IQ matched controls participated in the experiment (see **Table 1** for descriptive statistics). Twenty students (10 with MLD and 10 matched controls) were recruited from the University of York and 12 participants (six with MLD and six matched controls) were recruited from the University of Leuven. A sample of participants was recruited in both the University of York and the University of Leuven since it was not possible to recruit enough students with MLD who showed a clear history of MLDs within one university.

**Table 1:** Descriptive statistics of the sample.

Group	*N*	Gender	Age (years)	Math achievement^a^	IQ^b^
***University of York***
MLD	10	8 females	22.98 (3.61)	13.10 (7.29)	68.20 (25.21)
Control	10	8 females	20.37 (2.70)	74.00 (19.14)	70.90 (24.72)
					
***University of Leuven***
MLD	6	8 females	21.21 (1.36)	21 (12.34)	58.33 (25.82)
Control	6	8 females	20.87 (1.77)	91 (8.18)	66.67 (12.91)

*Note. Standard deviations are presented in parentheses.*

a Mean percentile score on the WRAT-3 arithmetic subtest (for the students from the University of York) and mean percentile score on the Arithmetic Tempo Test (for the students from the University of Leuven).

b Mean percentile score on the WASI (for the students from the University of York) and mean percentile score on the Advanced Progressive Matrices (for the students from the University of Leuven).

#### Sample of the University of York

One of the students from the MLD group recruited at the University of York had a formal diagnosis of MLD, whereas the other nine were recruited on the basis of significant self-reported difficulties with mathematics (using the screening survey of Chinn, 2007). In addition, because most students did not have a formal diagnosis, a significant discrepancy between their performance on a standardized math achievement test [i.e., Wide Range Achievement Test (WRAT-3) arithmetic, Wilkinson, 1993] and their cognitive abilities needed to be present. The MLD group from the University of York had a WRAT 3-Arithmetic subtest (Wilkinson, 1993) score below the 25th percentile based on the population sample mean (individual scores ranging from 3 to 23), whereas the control group scored above the 25th percentile (individual scores ranging from 47 to 95). *T*-tests revealed that the MLD group had a significant lower mathematical ability than the control group [*t*(18) = -9.403, *p* < 0.0001]. No significant difference in age [*t*(18) = 1.881, *p* = 0.076] and IQ [*t*(18) = -0.217, *p* = 0.871] was observed between both groups.

#### Sample of the University of Leuven

All students from the MLD group recruited at the University of Leuven were previously formally diagnosed with MLD as a child (received after multidisciplinary assessment). Their diagnosis was recently (i.e., in the past 12 months) confirmed at the university’s assessment service. Due to time constraints and given that their formal diagnosis of MLD was recently confirmed, we did not administer a broad mathematics achievement and IQ test as we did in sample of participants from the university of York.^1^ Nevertheless, to be able to compare the performance of these students with the performance of a control group, we administered a short timed arithmetic test (de Vos, 1992) and the Advanced Progressive Matrices (APM, Raven, 1962). The timed arithmetic test is designed to assess arithmetic retrieval in elementary school and has norms available till sixth grade. Compared to the norms of 6th grade in elementary school, the students with MLD scored below the 25th percentile on the timed arithmetic test (mean percentile score = 21). It could thus be expected that they perform worse than the 25th percentile on a age-adequate test. The control participants had a mean percentile score of 91. *T*-tests revealed that the MLD group performed significantly worse as opposed to the control group on the timed arithmetic test [*t*(10) = -11.663, *p* < .0001]. The groups did not differ in age (*t *< 1) and non-verbal IQ (*t* < 1) (see **Table 1**).

### MATERIALS

#### Experimental tasks

***Symbolic and non-symbolic priming task.*** Stimuli were presented at the center of the screen in white on a black background using E-Prime 2.0 (Psychology Software Tools). The symbolic stimuli consisted of digits ranging from 1 to 9 (Arial font size 20) and the non-symbolic stimuli consisted of arrays of 1 to 9 dots. The stimuli were generated using the program developed by Gebuis and Reynvoet (2011), which manipulates five visual properties: (1) the convex hull (i.e., smallest contour around the array of dots), (2) the aggregate surface of the dots, (3) density (i.e., the aggregate surface divided by the convex hull), (4) the average diameter, and (5) contour length. The different visual cues of the stimuli co-varied positively with numerosity in half of the trials and negatively with numerosity in the other half. Subjects sat at approximately 50 cm from the screen.

Prime-target pairs were created by combining primes ranging from 1 to 9 (except 5) and targets 1, 4, 6, and 9, resulting in 32 (eight primes × four targets) possible prime-target combinations. Subjects were instructed not to respond to the prime and to classify the targets as smaller or larger than 5. On half of these trials prime and target elicited the same response (e.g., 2-1; congruent trials), whereas on the other half prime and target elicited different responses (i.e., 7-1; incongruent trials). Congruent trials were presented twice in each block, resulting in 48 trials per block (32 congruent trials and 16 incongruent trials). This was done because the PDE is computed on the basis of the congruent trials only (see also Reynvoet et al., 2009; Defever et al., 2011). The response incongruent trials lead to a response interference effect that masks the numerical distance effect and are therefore excluded from the analyses (e.g., Reynvoet et al., 2002). In total, subjects completed four blocks (i.e., 192 trials).

The symbolic and non-symbolic conditions were counterbalanced and each subject was randomly assigned to a condition order. Before each experiment started, five random practice trials from the list were presented. During the practice, feedback was provided. The sequence of a trial was as follows: a fixation-cross (500 ms), the prime (150 ms), a blank screen (50 ms), the target stimulus (until a response) and a blank screen (1200 ms). In total, the experiment took approximately 60 min. Breaks between the different conditions were provided to prevent fatigue.

#### Standardized tests

***Arithmetical ability.*** All participants from the University of York were tested on the arithmetic subtest of the WRAT – third edition (Wilkinson, 1993). This tests assesses the ability to count, read symbols, solve oral problems, and perform computations within a time-limit of 15 min.. The participants from the University of Leuven were tested with the Tempo-Test-Rekenen (i.e., Tempo test Arithmetic; de Vos, 1992). This test consists of five subtests: one for each type of operation (addition, subtraction, multiplication, and division) and one with mixed operations. Forty items of increasing difficulty are presented in each subtest and participants have 1 min to solve as many problems as possible.

***Intelligence test.*** The cognitive abilities of the participants recruited at the University of York were estimated using the Wechsler Abbreviated Scale of Intelligence (WASI; Wechsler, 1999). The participants with MLD were tested on all four subtests (Vocabulary, Block Design, Similarities, Matrix Reasoning)^2^; for the controls we estimated their cognitive ability based on their performance on two subtests (Vocabulary, Matrix Reasoning). This took approximately 40 to 60 min. The participants from the University of Leuven completed the APM (Raven, 1962) as a measure of intellectual ability.

Participants were tested in two sessions. In one session the priming experiments were carried out, whereas in the other session the arithmetical ability and intelligence tests were administered. Each session took place on a separate day and both were completed within 2 weeks. Participants gave informed written consent and were reimbursed for time and travel. The study had ethical approval from the Ethics Committee of the Department of Psychology, University of York.

## DATA ANALYSES

The PDE was examined by analyzing the performance on the targets for congruent trials only, as a function of the numerical distance between prime and target (see also Reynvoet et al., 2009; Defever et al., 2011). As previously mentioned, the response incongruent trials (i.e., 33%) lead to a response interference effect that masks the numerical distance effect (e.g., Reynvoet et al., 2002). All trials on which the prime and target were identical (i.e., 17%) were also excluded to prevent a visual repetition priming effect in the symbolic task. To compare the performance between groups, median RTs^3^ for correct responses on the targets and the error rates were analyzed and were entered separately in a repeated measures analysis of variance (ANOVA) with stimulus notation (symbolic or non-symbolic) and prime-target distance (1, 2, or 3) as within-participant factors and group (MLD or control group) as a between-participant factor. We computed the 95% confidence intervals (CIs) for all effects according to the formulas reported in Jarmasz and Hollands (2009, p. 130) using the program MorePower 6.0 (Campbell and Thompson, 2012). The CIs provide information about the lowest and highest mean values that might exist at the population level. The difference between two means will be significant if it is greater than the CI’s margin of error (i.e., half of the width of the CI) multiplied by √2 (Jarmasz and Hollands, 2009; Loftus and Masson, 1994; Campbell and Thompson, 2012). For example, a CI of ± 10 ms would suggest that our experiment had enough power to detect a difference between means of 14 ms or larger (CI* √2).

## RESULTS

### REACTION TIMES

Mauchly’s test of sphericity indicated that the assumption of sphericity had not been violated, χ^2^(2) = 0.968, *p* = 0.616 for distance, nor for the interaction between notation and distance, χ^2^(2) = 2.829, *p* = 0.243. The repeated measures ANOVA revealed no effect of group, *F* < 1, $\eta_{p}^{2}$ = 0.023, 95% CI ± 42 ms. A significant effect of prime-target distance was present, *F*(2,29) = 7.926, *p* < 0.010, $\eta_{p}^{2}$ = 0.353. The RTs increased with increasing prime-target distance as shown by a significant linear trend for prime-target distance, *F*(1,30) = 16.087, *p* < 0.0001, $\eta_{p}^{2}$ = 0*.*349, 95% CI ± 6 ms (see **Table 2**). There was also a significant effect of stimulus notation, *F*(1,30) = 16.018, *p* < 0.0001, $\eta_{p}^{2}$ = 0*.*348, 95% CI ± 18 ms, indicating that responses were faster in the symbolic task (i.e., 501 ms) compared to the non-symbolic task (i.e., 551 ms). The two-way interactions between stimulus notation and group, *F* < 1, $\eta_{p}^{2}$ = 0.012, 95% CI ± 45 ms, between prime-target distance and group, *F*(2,29) = 1.711, *p* = 0.198, $\eta_{p}^{2}$ = 0.106 and between stimulus notation and prime-target distance, *F* < 1, $\eta_{p}^{2}$ = 0.064, 95% CI ± 10 ms, were not significant nor was the three-way interaction between stimulus notation, prime-target distance and group, *F* < 1, $\eta_{p}^{2}$ = 0*.*041, 95% CI ± 43 ms.

**Table 2:** Mean percentage error rates and reaction times (standard deviations) on the targets as a function of prime-target distance.

	Prime-target distance					
	Symbolic condition			Non-symbolic condition		
	1	2	3	1	2	3
***Error rates (%)***
MLD	1.31 (2.68)	3.56 (3.83)	2.81 (2.79)	6.31 (7.84)	5.69 (5.17)	6.88 (5.84)
Control	2.44 (3.32)	2.25 (2.79)	1.31 (2.18)	4.31 (3.95)	4.88 (6.56)	7.56 (7.06)
***RTs (ms)***
MLD	510 (101.39)	519 (109.89)	522 (131.06)	543 (102.67)	559 (96.07)	578 (104.72)
Control	479 (55.62)	483 (57.13)	493 (40.15)	541 (85.56)	534 (83.42)	553 (92.13)

### ERROR RATES

Mauchly’s test of sphericity indicated that the assumption of sphericity had not been violated, χ^2^(2) = 0.088, *p* = 0.957 for distance, nor for the interaction between notation and distance, χ^2^(2) = 1.928, *p* = 0.381. The repeated measures ANOVA revealed no effect of group, *F* < 1, $\eta_{p}^{2}$ = .0010, 95% CI ± 1.7%. There was also no effect of prime-target distance, *F*(2,29) = 1.564, *p* = 0.226, $\eta_{p}^{2}$ = 0.097, 95% CI ± 0.8%. The significant effect of stimulus notation, *F*(1,30) = 17.512, *p* < 0.001, $\eta_{p}^{2}$ = 0.369, 95% CI ± 1.3%. indicated that more mistakes were made in the non-symbolic task (i.e., 5.9%) as opposed to the symbolic task (i.e., 2.3%). The two-way interaction between stimulus notation and group, *F* < 1, $\eta_{p}^{2}$ = 0.001, 95% CI ± 0.6%, prime-target distance and group, *F* < 1, $\eta_{p}^{2}$ = 0.014, 95%, CI ± 0.3%, and stimulus notation and prime-target distance, *F*(2,29) = 2.830, *p* = 0.075, $\eta_{p}^{2}$ = 0.163, CI ± 1.1%, were not significant. Finally, a significant three-way interaction between stimulus notation, prime-target distance and group was observed, *F*(1,30) = 3.572, *p* < 0.050, $\eta_{p}^{2}$ = 0.198, CI ± 9.4%, Pairwise comparisons showed that only for distances 1 and 3, the MLD group made significantly more mistakes on the non-symbolic compared to the symbolic task, both *p*s < 0.024. The control group only made significantly more mistakes in the non-symbolic compared to the symbolic task for distance 3, *p* = 0.001. No significant differences between the MLD and control group for each distance condition in the symbolic, all *p*s > 0.100, and non-symbolic task, all *p*s > 0.369, were present.

## DISCUSSION

This study aimed at contrasting two hypotheses specifying the domain-specific deficit of magnitude representation underlying MLD. More specifically, we examined whether adults with MLD have (a) a deficit in the basic ability to represent numerosities, leading to difficulties in processing both symbolic and non-symbolic numerical magnitudes (i.e., representation deficit hypothesis; Dehaene, 1997; Butterworth, 2005) or (b) problems in accessing the number magnitude from symbols (i.e., access deficit hypothesis; Rousselle and Noël, 2007). To this purpose, the performance of adults with MLD and controls matched on age, gender and IQ was compared during a symbolic and non-symbolic priming task.

Our results showed that the size of the symbolic and non-symbolic PDE was comparable for adults with and without MLD. This finding is not in line with the representation deficit hypothesis, as it indicates the presence of a similar magnitude representation in both groups. According to the representation deficit hypothesis, a different PDE is expected for the MLD group compared to the control group because this would indicate differences in the precision of the magnitude representation. The conclusion of a similar magnitude representation in both groups is not in line with a previous study which provided evidence for the representation deficit hypothesis in adults with MLD (e.g., Mejias et al., 2012). These authors contrasted the performance of adults with and without MLD in both symbolic and non-symbolic estimation tasks. It was found that in all tasks, the estimates of adults with MLD were more variable and were less precise than those of the control participants, suggesting that adults with MLD demonstrate a less precise magnitude representation (i.e., representation deficit). The contrasting findings between the present study and the study of Mejias et al. (2012) might be due to differences in the number range. We only used numerosities ranging between 1 and 9, whereas Mejias et al. (2012) mainly used larger numerosities (i.e., 8–64). Undoubtedly, adults have a lot of experience with the numerical magnitudes 1 to 9. It has been suggested that individual’s experience shape their representation: the greater the experience, the more precise their processing (e.g., Lipton and Spelke, 2005; Verguts et al., 2005; Siegler and Opfer, 2003). Hence, the larger familiarity might have caused a similar performance between adults with MLD and control participants. A different pattern of performance between MLD and control participants might be found when a larger number range would be included in the priming task. It could also be wondered whether the difference in automaticity between our priming task and the estimation can explain the contrasting findings between our study and the one of Mejias et al. (2012). Indeed, researchers have shown that automatic and intentional number processing tasks can lead to different inferences about the magnitude representation (Cohen Kadosh and Walsh, 2009; Bugden and Ansari, 2011). Nevertheless, it should be noted that contrasting findings regarding the representation deficit hypothesis obtained with intentional number processing tasks are also observed in children with MLD (see De Smedt et al., 2013 for a review). Whereas some authors find worse performance on intentional non-symbolic comparison tasks in children with MLD compared to controls (e.g., Piazza et al., 2010; Mazzocco et al., 2011), others failed to find differences (e.g., Rousselle and Noël, 2007; Iuculano et al., 2008; Landerl and Kölle, 2009; De Smedt and Gilmore, 2011). Though, in the latter studies, children with MLD showed significant problems in the symbolic comparison task. Recently, authors suggested that a poorer inhibitory capacity in children with MLD might cause differences between MLD and controls on non-symbolic comparison tasks, rather than a representation deficit (Szücs et al., 2013). This is explained by the fact that the incongruent trials in a comparison task, in which visual cues are manipulated incongruently with numerosity, require participants to inhibit irrelevant visual cues (Gilmore et al., 2013). Future studies examining the representation deficit hypothesis in children and adults should thus include the assessment of general cognitive abilities. Clearly, to date, there is no robust evidence for a deficient magnitude representation causing MLD which is in contrast to the robust and ever-occurring evidence for the access deficit hypothesis (e.g., Landerl and Kölle, 2009; De Smedt and Gilmore, 2011). In line with this, it might be questioned whether the difficulties of participants with MLD in an estimation task with large numerical magnitudes are due to difficulties with the symbolic component of the task. Indeed, a symbolic estimation task involves a mapping between a symbol and its non-symbolic representation and it cannot be excluded that this is the case for the non-symbolic task as well. In the non-symbolic estimation task, participants might first translate the target numerosity into a symbol before reproducing an amount of dots. It is thus possible that adults with MLD performed worse than controls on the estimation tasks due to problems in processing large numerical symbols. To enhance our understanding of the specific difficulties in adults with MLD, it would be interesting for future studies to examine both the processing of large symbolic and non-symbolic numerical magnitudes using tasks which are purely symbolic and non-symbolic.

Our results also did not provide support for the access deficit hypothesis in adults. No evidence for difficulties with the processing of the symbols 1–9 was observed: the participants with MLD were not significantly slower than controls in the symbolic task. Similar to the controls, adults with MLD showed faster RTs on the symbolic compared to the non-symbolic priming task. This suggests that adults with MLD do not have difficulties in linking the symbols 1 to 9 with their corresponding magnitude representation, which is in contrast with findings reported in children with MLD. Consistent and ever-occurring difficulties in processing numerical symbols 1–9 have been found in children with MLD (see for a review, De Smedt et al., 2013). Our data suggests that this deficit in the basic processing of the symbols 1–9 which is experienced by children with MLD is resolved by or compensated for in adulthood. Nevertheless, severe difficulties in higher-level number processing are still present in the adults with MLD, as indicated by their persistent struggle with mathematics (e.g., extremely low scores on the more complex mathematics achievement tests). Although difficulties with the basic processing of relatively small numerical symbols might be remediated by adulthood, such difficulties during childhood might be a stumble block for acquiring more complex mathematical abilities. It should be acknowledged that the small sample size in our study requires careful interpretation of our findings. Although not significant, the difference in RT between the MLD and control group for the symbolic task was larger (i.e., 32 ms) compared to the difference in the non-symbolic task (i.e., 18 ms). The standard deviations around the means were substantially larger in the MLD group than in the control group, especially for the symbolic priming task. This might indicate large inter-individual differences in the MLD group in the ability to process numerical symbols 1–9. A replication of our study using a larger sample size is required to verify whether significant differences might be revealed. Moreover, longitudinal studies are needed to reveal whether training on numerical symbols in children with MLD have a positive impact on the acquisition of higher-level mathematics skills.

## Conflict of Interest Statement

The authors declare that the research was conducted in the absence of any commercial or financial relationships that could be construed as a potential conflict of interest.

## Footnotes

1 We verified whether differences in results could be found between the sample of participants with MLD from the University of York and the sample of participants with MLD from the University of Leuven. To this end, we submitted the RTs and error rates to a repeated measures ANOVA with notation (symbolic and non-symbolic) and distance (d1, d2, and d3) as within-subject factors and group (sample from the University of York and sample from the University of Leuven) as a between-subject factor. The RT analysis revealed no main effect of group (*F* < 1), nor were there any other significant interactions involving group (all *p*s > 0.332). Similarly, in the error rate analysis, no effect of group (*p* = 0.157) nor significant interactions involving group were observed (all *p*s > 0.140). Therefore, we did not consider them as two separate groups in the analyses.

2 We computed a two subtest-IQ for the participants with MLD, to verify whether this would differ from their four subtest-IQ: no significant difference was found (*t* < 1).

3 For each participant, we calculated the median RT per prime-target condition. We did not use a particular cut-off criterion (removal of trials on which the RT is below or above a certain threshold), since it is difficult to determine a priori which cutoff should be used. Therefore, we opted to use median RTs because it has the important advantage that they are insensitive to extreme values.
